# An Interpretable Framework for Identifying EEG Spectral Biomarkers of Pathological Brain Aging

**DOI:** 10.3390/diagnostics16193143

**Published:** 2026-09-27

**Authors:** Yunier Prieur-Coloma, Pavel Prado, Wael El-Deredy, Alejandro Weinstein

**Affiliations:** 1 Advanced Center for Electrical and Electronic Engineering, Universidad Técnica Federico Santa María, Valparaíso 2340000, Chile; yunier.prieur@usm.cl; 2 ValgrAI-Valencian Graduate School and Research Network for Artificial Intelligence, 46022 Valencia, Spain; wael.el-deredy@uv.cl; 3 Facultad de Ciencias de la Rehabilitación y Calidad de Vida, Universidad San Sebastián, Santiago de Chile 8420524, Chile; pavel.prado@uss.cl; 4 Escuela de Ingeniería Civil Biomédica, Universidad de Valparaíso, Valparaíso 2340000, Chile; 5 Department of Electronic Engineering, Universidad Técnica Federico Santa María, Valparaíso 2340000, Chile

**Keywords:** electroencephalography, explainable machine learning, EEG biomarkers, brain aging, neurodegenerative disorders

## Abstract

**Background**: Normal aging involves gradual declines in brain function, whereas neurodegenerative disorders show accelerated deterioration. Electroencephalography (EEG) captures alterations in brain oscillatory activity, making it promising for detecting neurodegenerative disorders. However, existing approaches often emphasize classification performance over spectral biomarker identification, limiting clinical translation. **Methods**: We propose an EEG-based framework integrating explainable machine learning, analyses of inter-subject heterogeneity and temporal stability, and probabilistic modeling to identify reproducible spectral biomarkers of pathological brain aging. Two independent public EEG datasets comprising cognitively normal (CN) individuals and patients with Alzheimer’s disease (AD), Parkinson’s disease (PD), and frontotemporal dementia (FTD) were analyzed. Spectral features from canonical frequency bands were analyzed using SHAP to identify reproducible biomarkers in two datasets. The identified biomarker was subsequently characterized through inter-subject heterogeneity, temporal stability, and empirical distributions to assess disease-related alterations in oscillatory activity. **Results**: Several spectral features showed consistent disease-related associations across classification tasks. The θ/α ratio emerged as a representative biomarker, showing reduced temporal stability and broader empirical distributions in most neurodegenerative groups than in CN. **Conclusions**: Reduced temporal stability of reproducible EEG spectral biomarkers may represent an observable signature of disrupted oscillatory brain organization during pathological aging. The proposed framework characterizes a dimension of EEG spectral biomarkers that static summaries discard, offering an interpretable basis on which future longitudinal studies may build diagnostic and monitoring tools.

## 1. Introduction

During aging, the human brain undergoes progressive structural and functional alterations that affect neural organization and cognitive performance [1,2]. Normal aging is typically associated with gradual changes in cognitive and motor functions, even in individuals without a clinical diagnosis [3,4]. Previous studies have shown that aging is accompanied by modifications in brain connectivity and oscillatory activity [5,6]. In contrast, neurodegenerative disorders such as Alzheimer’s disease (AD) and other dementias are associated with accelerated cognitive decline and disruption of brain function beyond that expected in age-matched healthy individuals [7]. Alterations in brain function occur years before the appearance of clinical symptoms [8]. Early detection during the preclinical stage provides a critical window of opportunity for implementing interventions that may slow disease progression and improve patients’ quality of life. Growing research efforts have focused on identifying electrophysiological signatures of neurodegenerative disorders in aging populations [9,10]. Electroencephalography (EEG) is particularly well suited for detecting alterations in oscillatory activity due to its non-invasive nature, wide accessibility, low cost, and high temporal resolution [11]. EEG has been widely employed for the diagnosis of neurodegenerative disorders, including AD [12], Parkinson’s disease (PD) [13], and Mild Cognitive Impairment (MCI) [14].

EEG-based machine learning (ML) and deep learning approaches for the detection of neurodegenerative diseases have gained increasing attention. EEG-based ML models typically rely on engineered spectral and functional descriptors of the brain activity to discriminate patients with neurodegenerative disorders from healthy controls. Early work by Fiscon et al. analyzed AD and MCI using frequency-domain features derived from fast Fourier transform and discrete wavelet transform coefficients across the canonical EEG frequency bands [15]. Using decision tree-based classifiers, the authors evaluated the discrimination of MCI and AD relative to cognitively normal (CN) subjects. The work proposed by Miltiadous et al. [16] used energy-based spectral features and statistical dispersion measures across the five canonical EEG bands to discriminate AD and frontotemporal dementia (FTD). Their study evaluated the performance of multiple classifiers, including decision trees, Random Forests, Support Vector Machines (SVMs), Naïve Bayes, and k-Nearest Neighbors (k-NN). More recent studies have emphasized the combination of complementary EEG spectral features and inter-band power ratios to improve the AD classification performance [17]. In parallel, other studies have shown alterations in EEG spectral features associated with PD [18]. Jaramillo-Jiménez et al. conducted a large multicenter study to characterize PD-related spectral changes [19]. Their results showed increased theta power in patients with PD. In [13], band power, variance, and entropy-based spectral features were used to discriminate PD in on- and off-medication states from CN. Multiple classifiers were evaluated, including Random Forests, Linear and Quadratic Discriminant Analysis, SVMs, and k-NN. However, these studies were focused on optimizing classification performance, rather than uncovering disease-specific spectral biomarkers.

Deep learning-based approaches learn hierarchical representations directly from EEG recordings and often improve classification performance [20]. However, their “black-box” nature has limited their clinical applicability. A critical step toward the clinical adoption of EEG-based diagnostic models is shifting the focus from maximizing classification performance to identifying physiologically meaningful EEG biomarkers of pathological brain aging. Previous work aimed at identifying EEG biomarkers has reported abnormal spectral patterns across multiple frequency bands [21,22]. These spectral hallmarks include spectral slowing, increased power in low-frequency EEG bands, and reduced power in higher-frequency oscillations [23,24]. Although these studies consistently report alterations in spectral activity during neurodegeneration, the identified biomarkers often vary across studies, limiting their reproducibility and clinical translation.

Neural compensation plays a central role in preserving brain function despite progressive structural deterioration. Compensatory neural mechanisms allow the aging brain to reorganize functional activity and maintain relatively stable cognitive performance during healthy aging and the early stages of neurodegeneration. As neurodegenerative processes progress, however, these compensatory mechanisms are believed to become insufficient, leading to a disruption of large-scale oscillatory organization and decreasingly stable neural activity [25]. From this perspective, changes in the temporal stability of EEG spectral biomarkers may reflect the progressive breakdown of compensatory capacity, thereby providing a complementary and clinically actionable signal that could support not only the discrimination between normal and pathological aging, but also the longitudinal monitoring of disease progression in at-risk individuals.

Despite previous advances, important challenges remain. First, few studies have identified consistent EEG biomarkers with discriminative behavior in different datasets acquired under distinct clinical and technical conditions. Second, although growing evidence suggests that neurodegeneration is accompanied by progressive disruption of brain oscillatory organization, whether these alterations are reflected in the temporal stability of EEG spectral biomarkers remains unexplored.

In this work, we propose an EEG-based framework for identifying and characterizing reproducible spectral biomarkers associated with pathological brain aging. First, explainable machine learning is employed to identify spectral biomarkers that consistently discriminate neurodegenerative conditions and to characterize the directionality of their contributions to disease classification. Subsequently, the identified biomarker is characterized in terms of inter-subject heterogeneity and temporal stability. Finally, probabilistic modeling is used to characterize its statistical organization across CN and neurodegenerative populations. The proposed framework is validated using two EEG datasets comprising older adults with normal aging and multiple neurodegenerative disorders. To the best of our knowledge, this is the first study to jointly identify reproducible EEG spectral biomarkers of neurodegeneration and characterize their temporal stability as a potential observable signature of disrupted oscillatory organization, providing an interpretable characterization of EEG spectral biomarkers on which future work on early diagnosis and longitudinal monitoring may build.

## 2. Methods

### 2.1. Framework Overview

In this work, we propose an EEG-based framework to identify and characterize consistent spectral biomarkers associated with neurodegenerative disorders. As summarized in Figure 1, the framework comprises the following stages: EEG preprocessing, spectral feature extraction, discrimination of neurodegenerative disorders, identification of spectral biomarkers, and statistical characterization of their inter-subject heterogeneity, temporal stability, and distributional organization. The details of each stage are described in the following sections.

### 2.2. EEG Preprocessing

EEG recordings are preprocessed following a standardized procedure designed to ensure signal comparability. First, signals are downsampled from their original sampling rate to 256 Hz in order to reduce computational complexity and storage requirements. Subsequently, all signals are standardized using a z-score transformation (zero mean and unit variance), applied independently to each EEG channel within each subject. Machine learning-based classification systems typically require a sufficiently large number of samples to achieve robust generalization. In EEG-based disorder classification, a common strategy is to segment continuous recordings into short, informative epochs, which increases the effective sample size while preserving the intrinsic characteristics of the signal. Following recent studies [26,27], EEG recordings are segmented into non-overlapping 4-s epochs to characterize spectral dynamics while preserving the signal’s intrinsic oscillatory properties. Although alternative epoch lengths may influence classification performance, the predefined 4-s epoch length is used throughout the analyses to provide a common setting for biomarker characterization rather than optimize classification performance.

### 2.3. Feature Extraction

Spectral features are extracted from each epoch utilizing statistical descriptors of relative band power and spectral entropy, which have been widely used in previous studies to discriminate neurodegenerative disorders from normal aging [16,28]. Relative band power (RBP) quantifies the proportion of signal power contained within a specific frequency band relative to the total power across the analyzed frequency range. The power spectral density (PSD) is estimated using Welch’s method with a Hamming window [29], with 1-s segments, 50% overlap, and a resulting frequency resolution of Δf=1 Hz. RBP is computed for the five canonical EEG frequency bands, delta δ (1–4 Hz), theta θ (4–8 Hz), alpha α (8–12 Hz), beta β (12–30 Hz), and gamma γ (30–40 Hz) [30], as follows:

(1) $${\mathrm{RBP}}_{b}^{\left(c\right)}=\frac{\sum_{f=f_{b}^{\min}}^{f_{b}^{\max}}{\mathrm{PSD}}_{c}\left(f\right)}{\sum_{f=f_{\mathrm{low}}}^{f_{\mathrm{high}}}{\mathrm{PSD}}_{c}\left(f\right)}$$

where ${\mathrm{PSD}}_{c}\left(f\right)$ denotes the power spectral density at frequency *f* for channel *c*, $f_{b}^{\min}$ and $f_{b}^{\max}$ define the frequency limits of band *b*, and $f_{\mathrm{low}}=1$ Hz and $f_{\mathrm{high}}=40$ Hz correspond to the minimum and maximum frequencies considered in the analysis, respectively. To summarize the distribution of RBP across EEG channels, statistical descriptors are computed for each frequency band from the RBP values across channels. Specifically, the mean, median, and standard deviation of the RBP are calculated across channels, providing complementary information on the central tendency and spatial variability of oscillatory activity within each EEG frequency band.

Spectral power ratios (SPRs) are computed to characterize the relative balance between slow and fast oscillatory components. SPRs are derived from the mean RBP values across EEG channels. For each epoch, an SPR is defined as follows:

(2) $${\mathrm{SPR}}_{b_{1}/b_{2}}=\frac{{\bar{\mathrm{RBP}}}_{b_{1}}}{{\bar{\mathrm{RBP}}}_{b_{2}}},$$

where $b_{1}$ and $b_{2}$ denote two distinct frequency bands and ${\bar{\mathrm{RBP}}}_{b}$ represents the mean relative band power across EEG channels for band *b*. The following SPRs are included: δ/θ, δ/α, δ/β, θ/α, θ/β, and α/β.

In addition to band-specific features, wide-band spectral entropy (SE) is computed across the full frequency range (1–40 Hz) to provide a global measure of spectral complexity. Spectral entropy quantifies the degree of irregularity and dispersion of spectral power across frequencies. SE is computed from the PSD averaged across EEG channels as follows:

(3) $$SE=-\sum_{j}p\left(f_{j}\right)\;\log\left(p(f_{j})\right),$$

where $p(f_{j})$ denotes the normalized channel-averaged PSD at the *j*-th frequency bin, defined as:

(4) $$p\left(f_{j}\right)=\frac{\mathrm{PSD}(f_{j})}{\sum_{j}\mathrm{PSD}\left(f_{j}\right)}.$$

In total, 22 spectral features are extracted from each epoch. These comprise statistical descriptors (mean, median, and standard deviation) computed for each canonical frequency band, inter-band SPRs computed from the mean relative band power, and wide-band spectral entropy.

### 2.4. Detection of Neurodegenerative Conditions

To evaluate the capacity of the spectral features for detection of pathological brain aging, binary classification models were constructed to discriminate subjects with a neurodegenerative condition from CN older adults. The labels are defined according to the available clinical diagnosis of each subject. A Random Forest (RF) classifier is employed, given its robust performance in recent EEG-based classification studies [31,32]. RF is an ensemble learning method that constructs multiple decision trees from bootstrap samples of the training data and combines their outputs through majority voting, thereby reducing variance and improving generalization. The RF classifier is parameterized with 200 trees, no predefined maximum depth, square-root feature sampling at each split, a minimum of two samples per split, and one sample per leaf. Class weights are determined using the balanced-subsample strategy, and the random seed is fixed to ensure reproducibility. The same parameter settings are applied in all classification tasks. The classifier is trained and validated using spectral features extracted from multiple epochs. Each epoch from a given subject represents one sample for the model. However, these samples are not independent (several segments are from the same subject). Commonly, epochs from the same subject are simultaneously included in both the training and testing sets (data leakage), thereby the model’s performance will be artificially inflated. We avoid this issue and ensure strict subject-level separation, using a Leave-One-Subject-Out Cross-Validation (LOSOCV) strategy [33]. In LOSOCV, all segments from a single subject are held out at each iteration, while the classifier is trained on the remaining subjects’ data. The trained model is then evaluated on the left-out subject’s epochs. This procedure is repeated for each subject, yielding an unbiased estimation of generalization performance to unseen subjects within each dataset. Note that model predictions are generated at the epoch level, whereas the final decision must be made at the subject level. Following the approach proposed by Sibilano et al. [34], subject-level predictions were obtained by averaging the predicted class probabilities across all epochs belonging to the same subject. The final class label was assigned according to the class with the highest mean probability.

Model performance was evaluated at the subject level using Accuracy, Sensitivity, Specificity, Precision, and F1-score. These metrics are computed from the confusion matrix, where true positives (TP) and true negatives (TN) denote the number of diseased and control subjects correctly classified, respectively. False positives (FP) are the number of control subjects classified as diseased. False negatives (FN) correspond to diseased subjects classified as control. Accuracy (Acc) measures the overall proportion of correctly classified subjects. Sensitivity (Sen) quantifies the model’s ability to correctly identify subjects with neurodegenerative conditions, whereas Specificity (Spe) measures its ability to correctly identify control subjects. Precision (Prec) reflects the proportion of subjects predicted as diseased that are positive. The F1-score (F1), as the harmonic mean of Precision and Sensitivity, provides a comprehensive measure of model robustness by balancing FP and FN, making it particularly informative when class distributions are imbalanced. The 95% confidence intervals (CI, 2.5th and 97.5th percentiles) are estimated for Acc, Sen, Spe, Prec, and F1 using stratified bootstrap resampling (1000 iterations) at the subject level, applied to the final out-of-subject predictions obtained from LOSOCV. The performance metrics are defined as follows:

(5) $$Acc=\frac{TP+TN}{TP+TN+FP+FN},$$

(6) $$Sen=\frac{TP}{TP+FN},$$

(7) $$Spe=\frac{TN}{TN+FP},$$

(8) $$Prec=\frac{TP}{TP+FP},$$

(9) $$F1=2\times \frac{Prec\times Sen}{Prec+Sen}.$$

The classification stage identifies candidate spectral biomarkers rather than estimating disease-specific effects. Demographic covariates are therefore excluded from the classification models.

### 2.5. Identification of Spectral Biomarkers

To facilitate clinical interpretation, explainable ML is employed to identify reproducible EEG spectral biomarkers that consistently distinguish neurodegenerative conditions from normal aging. Spectral features showing consistent discriminative relevance and directionality across independently fitted models are considered reproducible candidate biomarkers. In particular, RF classification is complemented with a SHAP-based explainability analysis to characterize the contribution of spectral features to model predictions. We use TreeSHAP, an efficient SHAP implementation specifically designed for tree-based models [35]. SHAP provides a unified game-theoretic framework for quantifying each feature’s contribution to an individual prediction. TreeSHAP computes Shapley values for ensemble tree models by leveraging the internal structure of the ensemble. A Shapley value represents the marginal contribution of a feature to a given prediction, considering all possible coalitions of features. For each LOSCOV fold, SHAP values are computed for epochs from the held-out subject. Positive SHAP values indicate that a feature pushes the model output toward the diseased condition, whereas negative values push the prediction toward the control class. The sign of the feature–SHAP correlation is used to characterize the direction of a feature’s contribution to model predictions. Using TreeSHAP thus enables a fine-grained, disorder-specific interpretability analysis that complements global model performance metrics and provides insights into the spectral features associated with neurodegenerative diseases [31].

### 2.6. Statistical Characterization of Spectral Biomarkers

Following the identification of reproducible EEG spectral biomarkers, we investigated whether pathological brain aging is associated with alterations in their variability and statistical organization. The identified biomarkers are characterized through three complementary analyses. First, inter-subject heterogeneity characterizes the variability of biomarker values across individuals within each group. Second, temporal stability quantifies fluctuations in biomarker values across consecutive epochs within each subject. Finally, distributional modeling characterizes the empirical distributions of these biomarkers, providing a statistical description of their organization across normal and pathological aging conditions.

#### 2.6.1. Inter-Subject Heterogeneity and Temporal Stability Analyses

According to recent works, neurodegeneration progressively overwhelms the brain’s compensatory mechanisms, resulting in a progressive disruption of oscillatory brain organization [25]. We therefore investigated whether the identified spectral biomarkers exhibit alterations in inter-subject heterogeneity and temporal stability associated with neurodegenerative conditions. Inter-subject heterogeneity is quantified as the standard deviation of biomarker values across subjects within each group at each epoch. Temporal stability is quantified by computing the interquartile range (IQR) of biomarker values across consecutive epochs within each subject’s resting-state recording. Thus, this measure characterizes absolute short-term fluctuations during a single recording. Pairwise differences in temporal stability between each disease condition and the corresponding CN group are evaluated using the Mann–Whitney U test. To test for potential age confounding, the pairwise comparisons are further assessed using analysis of covariance (ANCOVA) [36]. Group is defined as the independent variable, Age is included as a covariate, and the IQR of each feature is used as the dependent variable. The slope homogeneity is assessed by including a Group × Age interaction term. For each term (Group, Age, and Group × Age), the *F* statistic is computed, and statistical significance is assessed using the associated *p*-value. Partial eta squared ($\eta_{p}^{2}$) is calculated as a measure of effect size. For these analyses, IQR values are log-transformed to reduce distributional asymmetry.

#### 2.6.2. Distributional Modeling

To characterize the distributional organization of the identified spectral biomarker two analyses are performed using epoch values of each group. First, candidate probability distributions are compared to assess their ability to describe the empirical data. Afterwards, based on these comparisons, a common reference model is retained to characterize and compare the biomarker distributions across groups within each dataset. These analyses pool epochs across subjects within each group and therefore do not separate within-subject fluctuations from between-subject heterogeneity.

For model selection, three candidate distributions are considered: the power-law, exponential, and lognormal models. Rather than relying on histogram-based representations, which are sensitive to binning parameters, distribution fitting was performed using maximum likelihood estimation following the framework proposed by Clauset et al. [37]. The lower bound $x_{\min}$ is estimated for each group, and the model fittings are restricted to observations greater than $x_{\min}$, ensuring that all candidate distributions were compared over the same support (see Supplementary Material, Section SB.1). The corresponding probability density functions are defined as follows:

Power-law:

(10) $$p_{\mathrm{pow}}\left(x\right)=\frac{\alpha -1}{x_{\min}}{\left(\frac{x}{x_{\min}}\right)}^{-\alpha },\;\alpha >1$$

where α is the scaling parameter.

Exponential:

(11) $$p_{\exp}\left(x\right)=\lambda \exp\left(-\lambda (x-x_{\min})\right),\;\lambda >0$$

where λ is the rate parameter.

Lognormal:

(12) $$p_{log}\left(x\right)=\frac{1}{x\sigma \sqrt{2\pi }}\frac{\exp\left(-\frac{{\left(\ln\left(x\right)-\mu \right)}^{2}}{2\sigma^{2}}\right)}{\left(1-\Phi \left(\frac{\ln(x_{\min})-\mu }{\sigma }\right)\right)},$$

where μ and σ are the mean and standard deviation of ln(x), respectively, and Φ(·) is the cumulative distribution function (CDF) of the standard normal distribution.

The lower bound, $x_{\min}$, is estimated following the procedure described in [37]. The optimal value x^min is chosen to minimize the distance between the empirical distribution and the fitted model for $x\geq x_{\min}$. The Kolmogorov–Smirnov distance is used as a nonparametric measure, quantifying the maximum distance between the CDFs of the empirical data and the fitted model, as follows:

(13) $$D=\max_{x\geq x_{\min}}\left|S(x)-P(x)\right|,$$

where S(x) denotes the empirical CDF of the data restricted to values $x\geq x_{\min}$, and P(x) denotes the CDF of the fitted model over the same range. The estimate x^min corresponds to the value of $x_{\min}$ that minimizes *D*.

Model selection is conducted using log-likelihood ratio (*R*) tests. Given two candidate models $M_{1}$ and $M_{2}$, the log-likelihood ratio is defined as follows:

(14) $$R=\log\frac{L(M_{1}|x)}{L(M_{2}|x)},$$

where $L(M_{i}|x)$ denotes the likelihood of model $M_{i}$ given the data. Positive values of *R* indicate a preference for model $M_{1}$ over $M_{2}$, while negative values indicate the opposite.

### 2.7. Framework Validation Using Multiple Datasets

The proposed framework is validated using two publicly available datasets comprising CN older adults and older adults with neurodegenerative disorders. The datasets differ in cohort composition, clinical characteristics, and technical acquisition parameters, including the number of electrodes, recording systems, and preprocessing pipelines. This heterogeneity enables assessment of the framework’s ability to identify and characterize consistent spectral features across independently fitted models under distinct experimental and clinical conditions. Details of each dataset, including demographic information and preprocessing-specific parameters, are provided in the next section.

## 3. Results

This section presents the validation results of the proposed framework on two independent EEG datasets (BrainLat and Thessaloniki datasets, described below) comprising CN older adults and older adults with neurodegenerative disorders. For both datasets, the framework is evaluated by identifying reproducible spectral biomarkers associated with neurodegenerative conditions, and analyzing their statistical properties. Data processing and statistical analyses were performed using Python 3.11 (Python Software Foundation, Wilmington, DE, USA). All codes and data necessary to reproduce the reported results are publicly available at Zenodo https://doi.org/10.5281/zenodo.22811463 (accessed on 23 September 2026) [38].

### 3.1. Datasets

BrainLat dataset: The BrainLat dataset comprises recordings from older adults with AD and Parkinson’s disease (PD), as well as CN [39]. The dataset provides resting-state EEG acquired using a 128-channel BioSemi ActiveTwo system (BioSemi B.V., Amsterdam, The Netherlands) equipped with pin-type active sintered Ag/AgCl electrodes. During EEG acquisition, participants remained with their eyes closed, and each recording lasted approximately 10 min. According to the dataset description, the signals were re-referenced to the common average, band-pass filtered between 0.5 and 40 Hz, and resampled to 512 Hz. EEG artifacts associated with eye movements and blinking were corrected using Independent Component Analysis (ICA). Regarding pharmacological treatment, PD patients were receiving levodopa and were evaluated during the on phase, further medication details were not available in the dataset description.

Thessaloniki dataset: The Thessaloniki dataset comprises EEG resting-state recordings from older adults with AD, frontotemporal dementia (FTD), and CN [16]. EEG signals correspond to resting-state recordings acquired using a Nihon Kohden EEG-2100 system (Nihon Kohden Corporation, Tokyo, Japan) with 19 scalp electrodes positioned according to the international 10–20 system. Recordings were performed with eyes closed, at a sampling rate of 500 Hz. According to the dataset documentation, preprocessing included re-referencing to the average reference and band-pass filtering between 0.5 and 45 Hz. Automatic artifact rejection was performed using Artifact Subspace Reconstruction (ASR), followed by ICA. Pharmacological treatment information was not reported in the dataset documentation.

Table 1 summarizes the clinical and demographic characteristics of both datasets.

For the BrainLat dataset, the segmentation procedure resulted in 2413, 3565, and 1739 epochs for the AD, PD, and CN groups, respectively. For the Thessaloniki dataset, a total of 7267, 4137, and 6015 epochs were obtained for the AD, FTD, and CN groups, respectively.

### 3.2. Neurodegenerative Detection in Two Datasets

Four disease classification tasks were performed in the two datasets: AD vs. CN and PD vs. CN in BrainLat, and AD vs. CN and FTD vs. CN in Thessaloniki. The classification tasks within each dataset shared the same CN group.Table 2 summarizes the classification performance across both datasets. For the BrainLat dataset, in the AD vs. CN scenario, the classifier achieved an accuracy of 67.7%[95% CI: 51.6–83.9%], indicating moderate discriminative power. The sensitivity was 73.6%[52.6–94.7%], suggesting that most AD subjects were correctly identified. In contrast, the PD vs. CN classification showed improved performance, with an accuracy of 82.5%[70.5–94.1%] and a sensitivity of 95.4%[86.4–100%], highlighting the model’s strong ability to detect PD cases. However, in both scenarios, specificity remained relatively low (58.3%), indicating a higher rate of false positives among CN subjects. Precision reached 73.6%[60.9–88.2%] for AD and 80.7%[71–91.7%] for PD, with corresponding F1-scores of 73.6%[57.1–87.2%] and 87.5%[80–95.5%], respectively.

For the Thessaloniki dataset, the AD vs. CN classification achieved an accuracy of 75.4%[64.6–86.2%], indicating a moderate performance. Sensitivity (75%[61.1–88.9%]) and specificity (75.8%[58.6–89.7%]) were well balanced, suggesting comparable reliability in identifying AD and CN subjects, respectively. Precision reached 79.4%[68.3–90.9%], with an F1-score of 77.1%[65.7–87.3%], reflecting a consistent performance across evaluation metrics. In the FTD vs. CN scenario, the classifier obtained an accuracy of 73.1%[61.5–82.7%]. In contrast to the AD case, specificity was notably higher (89.7%[75.9–100%]), indicating a strong ability to correctly identify CN subjects, whereas sensitivity was lower (52.2%[30.4–73.9%]), suggesting a reduced capability to detect FTD cases. Precision remained relatively high (80% [61.1–100%]), with an F1-score of 63.2%.

The confidence intervals reflect meaningful uncertainty in the performance estimates, consistent with the modest cohort size.

### 3.3. Reproducible Spectral Biomarkers in Two Datasets

Figure 2 presents the SHAP-based feature relevance for the positive class (e.g., diseased group) across all classification tasks for both datasets. Each panel displays the contributions of the top 11 spectral features, ranked independently by their SHAP importance within each task, along with their directional influence on the model predictions (see Supplementary Material, Figure S2, for the complete set of features). For the BrainLat dataset, features associated with the θ and α bands emerged as the most influential predictors in both classification tasks (Figure 2a,b). A similar pattern was observed in the Thessaloniki dataset for the AD vs. CN task (Figure 2c), where the classification was primarily driven by α and θ band-related features. In contrast, as shown in Figure 2d, the FTD vs. CN task was dominated by features associated with the β, γ, and α bands.

Figure 3 presents the feature-SHAP correlations between feature values and their corresponding SHAP values across all samples for each classification task and dataset. Positive correlations indicate that higher feature values drive model predictions toward the pathological class, whereas negative correlations indicate that higher feature values favor the control class. Several features exhibited consistently positive feature–SHAP correlations across all classification tasks and datasets, supporting their role as reproducible candidate spectral biomarkers of neurodegenerative conditions. Among these features, the θ/α ratio showed consistent discriminative relevance and directionality across independently fitted models (see Figure 3, black box) and was therefore selected as a representative biomarker for subsequent inter-subject heterogeneity, temporal stability, and distributional analyses.

### 3.4. Inter-Subject Heterogeneity and Temporal Stability of Representative Biomarker

Figure 4illustrates the inter-subject heterogeneity of the θ/α ratio across consecutive epochs for all classification tasks and datasets. In particular, disease groups exhibited larger inter-subject heterogeneity than CN subjects, reflected by wider ±1 standard deviation bands across epochs. Interestingly, a spectral feature with lower SHAP relevance that did not meet the reproducibility criterion did not exhibit a distinct variability pattern between conditions (see Supplementary Material, Figure S3).

Figure 5 shows the pairwise comparisons in temporal stability of the θ/α ratio between each neurodegenerative condition and the corresponding CN group for each dataset. The *p*-values from the pairwise Mann–Whitney U tests were adjusted within each dataset using the Holm method. Significant differences between disease and CN groups were observed in the BrainLat dataset for both AD vs. CN (p<0.05) and PD vs. CN (p<0.001) comparisons. Similarly, significant differences were found in the Thessaloniki dataset for AD vs. CN comparison (p<0.01), whereas no statistically significant differences were found for the FTD vs. CN comparison.

As shown in Table 3, the main effect of Group on temporal stability of the θ/α ratio remained significant after age adjustment for AD vs. CN and PD vs. CN in the BrainLat dataset, and for AD vs. CN in the Thessaloniki dataset, whereas no significant Group effect was observed for FTD vs. CN. The effect of Age was significant only for PD vs. CN, while the Group×Age interaction was not statistically significant in any comparison.

### 3.5. Distributional Organization of Spectral Biomarker

Table 4 presents the model comparisons for the θ/α ratio biomarker across datasets and conditions. The power-law model was consistently disfavored relative to both lognormal and exponential models. Comparisons between the lognormal and exponential models showed specific patterns for each condition. In Thessaloniki, the lognormal model was preferred across all groups. In BrainLat, the exponential model was preferred for AD, the lognormal model was preferred for PD, and no significant preference was observed for CN. No single distribution provided superior fit across all comparisons. Accordingly, the lognormal distribution was retained as a convenient common descriptive model for comparing biomarker distributions across datasets and conditions. This selection was made for descriptive reference purposes rather than on the basis of statistical superiority. The lognormal model was therefore fitted to the complete positive empirical distribution of each group.

Figure 6 shows the lognormal models fitted to the complete positive empirical distributions of the θ/α ratio across datasets and conditions. In all groups, the distributions are positively skewed and heavy-tailed, departing markedly from a Gaussian shape. Because these fits pool epochs across subjects, we do not interpret differences in width and location between groups as group-level effects. The Supplementary Material (Table S1) reports the fitted model parameters for each dataset.

## 4. Discussion

In this study, we proposed an EEG-based framework for identifying and characterizing spectral biomarkers associated with pathological brain aging. By integrating explainable machine learning, inter-subject heterogeneity and temporal stability analyses, and probabilistic modeling, the proposed approach provides a comprehensive characterization of disease-related alterations in EEG spectral dynamics.

Aging induces progressive structural and functional alterations in the human brain, requiring adaptive and compensatory mechanisms to preserve cognitive and neural function. The brain’s adaptive capacity may partially compensate for early pathological alterations, thereby masking disease-related signatures. However, as neural dysfunction progresses, compensatory capacity gradually becomes insufficient, leading to increasingly unstable neural activity. Within this context, our approach was designed to characterize changes in the temporal stability of EEG spectral biomarkers associated with neurodegeneration. Although the underlying compensatory mechanisms were not directly assessed, such changes may provide observable signatures of alterations in the processes responsible for preserving stable oscillatory organization.

Previous EEG studies have demonstrated that spectral features contain relevant information for distinguishing CN individuals from patients with neurodegenerative disorders [40]. ML-based approaches have combined band-power measures, spectral ratios, entropy, and other frequency-domain descriptors to detect AD, MCI, FTD, and PD [41]. However, most of these studies have primarily focused on maximizing classification performance, giving less attention to interpretable and reproducible spectral biomarkers underlying model predictions. Other studies have investigated EEG spectral biomarkers of neurodegeneration, consistently reporting spectral slowing, increased low-frequency activity, and reduced alpha- or higher-frequency power [42,43]. Nevertheless, the specific biomarkers identified vary considerably across studies and clinical populations, limiting their reproducibility and clinical applicability. The present framework addresses these limitations through three complementary components. First, it identifies reproducible EEG spectral biomarkers associated with neurodegeneration whose discriminative relevance and direction of association remain preserved across independent datasets acquired under different clinical and technical conditions. Second, it extends their characterization beyond discriminative by assessing inter-subject heterogeneity and temporal stability. Finally, probabilistic modeling provides a complementary description of their statistical organization across CN and neurodegenerative populations.

The proposed framework was validated using two public EEG datasets comprising CN older adults and subjects with highly prevalent neurodegenerative disorders, including AD, PD, and FTD. Across both cohorts, the framework successfully discriminated neurodegenerative conditions from CN aging, achieving classification performance comparable to previous EEG-based studies [44,45]. The classification stage was primarily used to identify disease-related spectral biomarkers; therefore, particular attention was given to model’s sensitivity. The lower sensitivity observed for FTD indicates a limited ability of the model to identify this condition. Since SHAP values are derived from the fitted classifier, the corresponding results may provide a less reliable characterization of the feature contributions associated with FTD. Unlike approaches primarily designed to maximize predictive accuracy, our framework was developed to balance classification performance with model interpretability, enabling the identification of physiologically meaningful spectral biomarkers.

We identified reproducible EEG spectral biomarkers whose discriminative relevance and direction of association were maintained despite differences in acquisition protocols and clinical populations. As a result, the θ/α ratio emerged as a representative biomarker, preserving its discriminative relevance across independent datasets and multiple neurodegenerative disorders. These findings extend previous evidence indicating that spectral power ratios constitute robust indicators of neurodegeneration [22]. Beyond its discriminative capability, the identified biomarker also exhibited distinct variability patterns in neurodegenerative conditions. At the inter-subject level, disease groups showed greater heterogeneity than CN older adults across both datasets. At the within-subject level, with the exception of the FTD group, neurodegenerative conditions exhibited lower temporal stability than CN individuals. These differences remained significant after age adjustment in the corresponding pairwise ANCOVA analyses. Although the underlying physiological mechanisms remain to be elucidated, these findings are consistent with recent work suggesting that neurodegeneration progressively disrupts the mechanisms responsible for maintaining the stable organization of brain function [46]. From a translational standpoint, the observed reduction in temporal stability suggests that longitudinal EEG biomarker assessment is worth evaluating for monitoring disease progression.

Probabilistic modeling further complemented these findings by characterizing the statistical organization of the identified biomarker. Neurodegenerative groups exhibited broader empirical distributions than CN individuals, consistent with greater inter-subject heterogeneity. Although no single probabilistic model was uniformly preferred across all datasets and conditions, the lognormal distribution provided a common descriptive representation of the evaluated biomarker, facilitating comparisons across neurodegenerative disorders and healthy aging. Taken together, these findings support the potential of EEG spectral biomarkers as a low-cost and non-invasive tool for characterizing pathological brain aging. Its consistency in two cohorts with distinct acquisition protocols suggests that this behavior is not specific to a single acquisition setting.

### Limitations and Future Work

Several methodological aspects should be considered. The EEG data were obtained from distinct studies employing different acquisition systems, spatial resolutions, and preprocessing pipelines, which may influence the spectral representation of EEG activity across datasets. In particular, the datasets differed substantially in the distribution of spectral power across EEG frequency bands. Similar limitations related to dataset heterogeneity have been reported in previous EEG studies involving neurodegenerative disorders [31]. Information on potential confounding factors was limited in the two public datasets. Education level was reported only for BrainLat, while information on vascular comorbidities was not available. Additionally, pharmacological information was not consistently available across datasets. Therefore, the potential influence of these demographic and clinical factors on EEG spectral features could not be excluded. Fixed frequency bands were used for all subjects to maintain spectral definitions across datasets and facilitate comparison with the broader EEG literature. However, fixed frequency bands may not capture individual shifts in oscillatory activity associated with aging and neurodegenerative disorders [47]. An alternative approach is to define individualized frequency bands based on subject-specific alpha peaks [48]. In practice, implementing this approach may introduce additional methodological challenges when reliable individual alpha peaks cannot be identified. The reported temporal variability was quantified within a single resting-state recording, and therefore characterizes short-term fluctuations of the biomarker rather than its stability over clinically meaningful time scales. Both datasets are cross-sectional and include cognitively normal participants and patients with established clinical diagnoses. The present results therefore do not establish utility for early screening, conversion prediction, or longitudinal disease monitoring. Evaluating screening and conversion prediction requires prospective follow-up of initially cognitively normal or at-risk individuals. Disease monitoring requires repeated EEG recordings linked to changes in clinical status, with assessment of both test–retest reliability and sensitivity to clinical change. Therefore, the present results do not support conclusions about the value of temporal variability as a preclinical marker. Testing this hypothesis requires repeated recordings of the same individuals, which would additionally allow us to estimate the test–retest reliability of the static and dynamic descriptors, a prerequisite for their use in longitudinal monitoring. Finally, we did not incorporate age into the classification or SHAP analyses, and the BrainLat groups differ in age. Consequently, the relevance attributed to the identified features may partly reflect age-related differences between groups.

## 5. Conclusions

This study presents an EEG-based framework for identifying and characterizing spectral biomarkers associated with pathological brain aging. Unlike conventional approaches focused primarily on classification performance, the proposed framework integrates explainable machine learning, inter-subject heterogeneity and temporal stability analyses, and probabilistic modeling to characterize EEG spectral biomarkers from complementary perspectives. Our results demonstrate that reproducible EEG spectral biomarkers can be consistently identified in two independent datasets. Furthermore, reduced temporal stability provides complementary information beyond conventional static spectral measures, suggesting that the dynamic behavior of spectral biomarkers offers an additional dimension for characterizing alterations associated with pathological brain aging. Overall, the proposed framework provides an interpretable methodology for studying EEG alterations across normal and pathological brain aging. By characterizing a dimension of EEG spectral biomarkers that static summaries discard, the proposed framework offers an interpretable basis for future longitudinal studies to build EEG-based tools for early screening and disease monitoring in clinical and community-based settings.

## Figures and Tables

**Figure 1:**
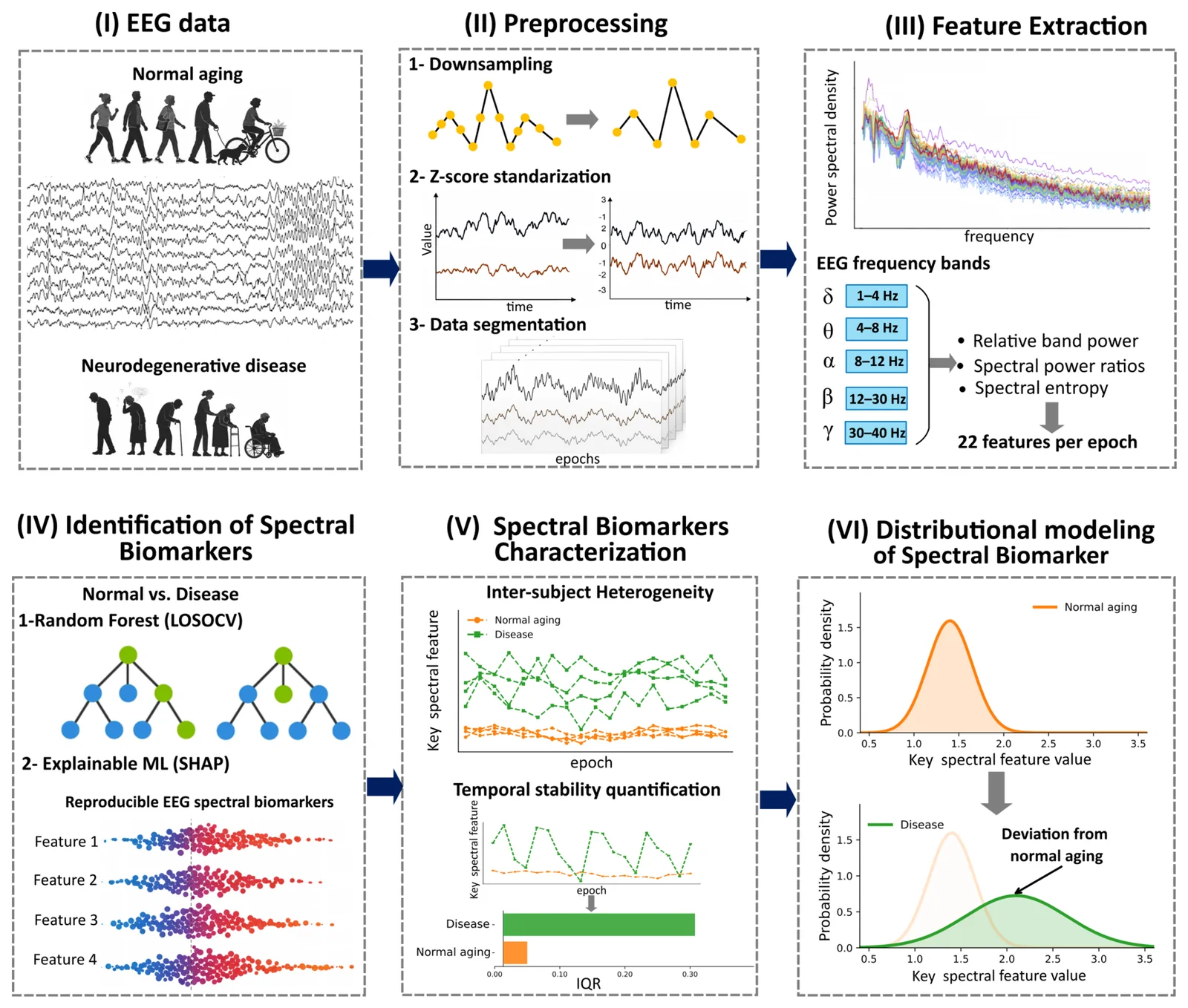
Overview of the proposed framework. (**I**) EEG recordings from CN older adults and individuals with neurodegenerative disorders are used. (**II**) Signals are preprocessed by downsampling, standardization, and epoch-based segmentation. (**III**) Spectral features are extracted from canonical EEG frequency bands. (**IV**) Explainable machine learning is employed to identify reproducible EEG spectral biomarkers that consistently distinguish neurodegenerative conditions from normal aging. (**V**) The identified biomarkers are characterized in terms of inter-subject heterogeneity and temporal stability. (**VI**) Distributional modeling is used to characterize the statistical organization of the identified spectral biomarkers in normal aging and neurodegenerative conditions.

**Figure 2:**
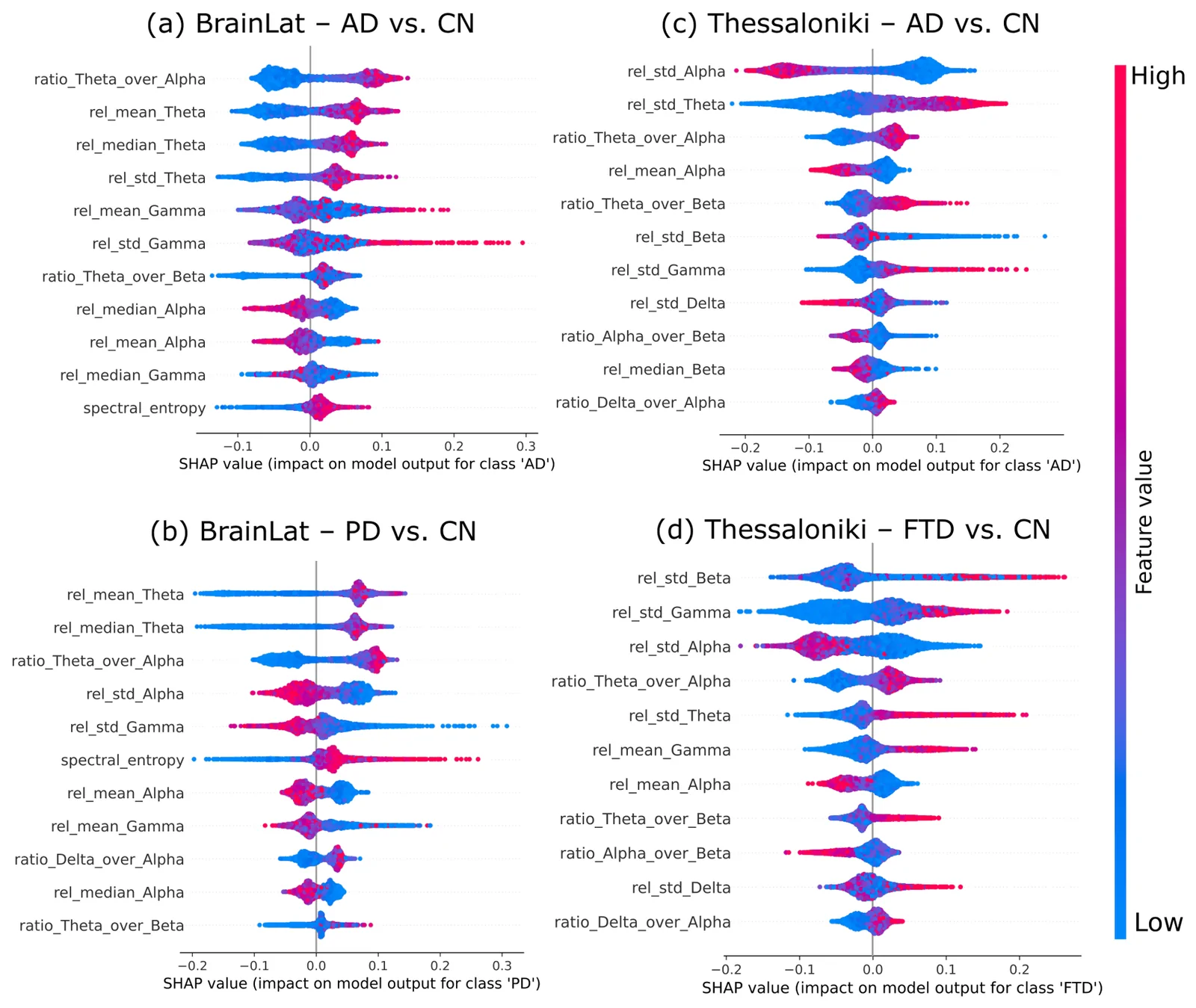
SHAP beeswarm plots for all dataset-specific classification tasks: (**a**) BrainLat—AD vs. CN; (**b**) BrainLat—PD vs. CN; (**c**) Thessaloniki—AD vs. CN; and (**d**) Thessaloniki—FTD vs. CN. Each panel shows the contribution and direction of influence of the top-ranked spectral features for the corresponding positive class. Features are ordered independently within each panel from highest to lowest relevance based on their SHAP importance, thereby highlighting the dominant discriminative signatures for each task.

**Figure 3:**
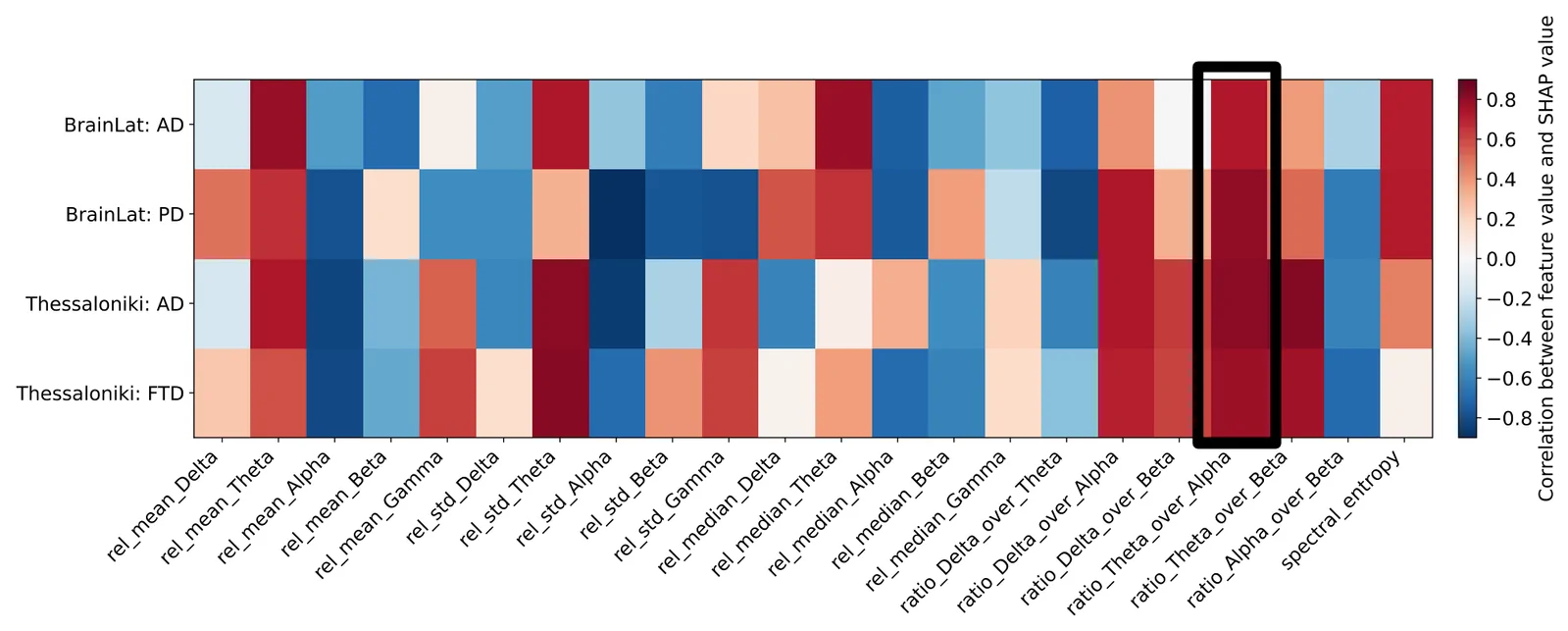
Feature–SHAP correlation analysis across two datasets and classification tasks. Rows correspond to disease-specific classification scenarios, and columns represent spectral features. Positive correlations indicate that higher feature values increase the model output toward the pathological class, whereas negative correlations indicate that higher feature values favor the control class. The highlighted region (black box) shows the consistent positive association of the θ/α ratio with neurodegenerative conditions in both datasets.

**Figure 4:**
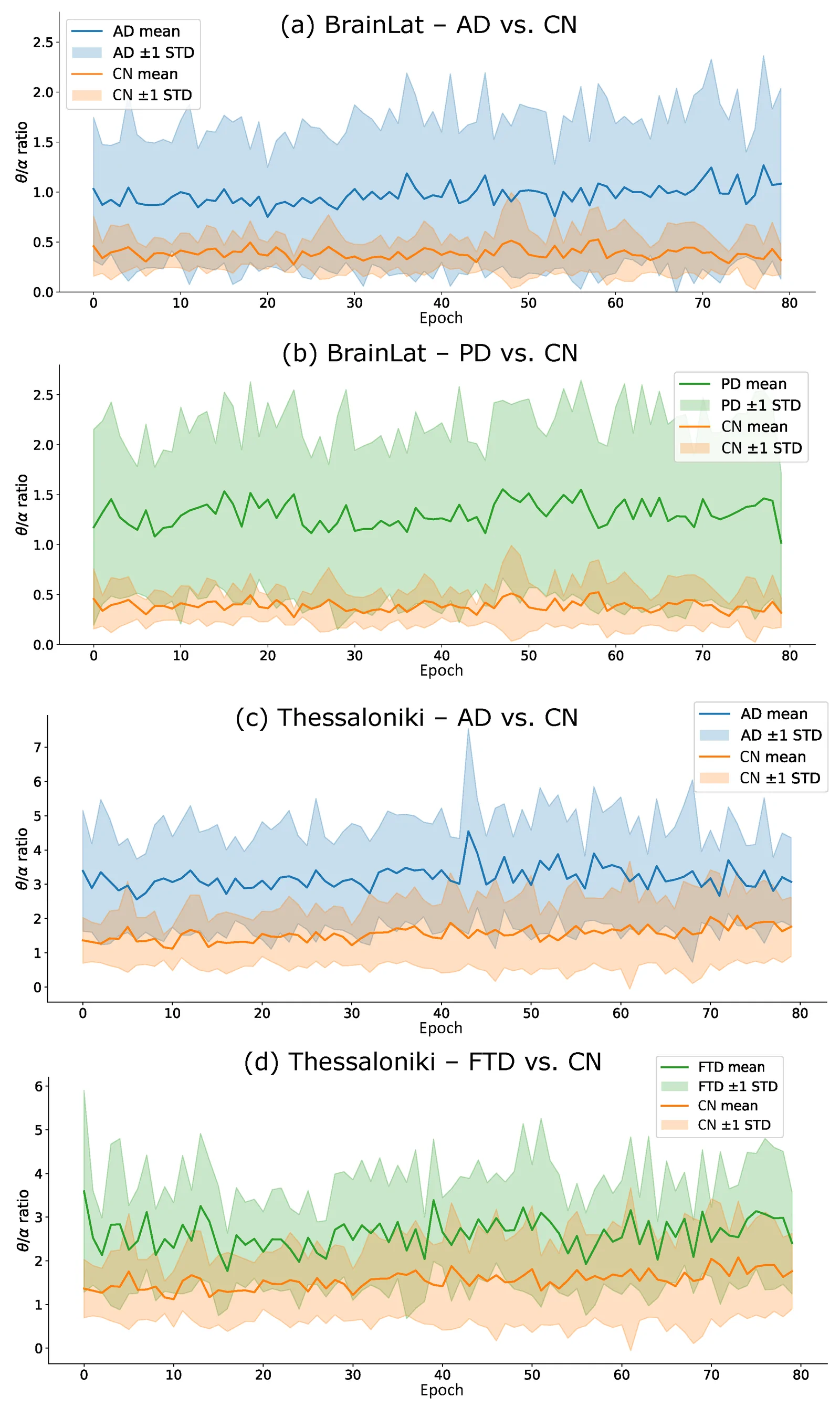
Evolution and inter-subject heterogeneity of the θ/α ratio across consecutive epochs for all classification tasks and datasets. Panels correspond to (**a**) BrainLat—AD vs. CN; (**b**) BrainLat—PD vs. CN; (**c**) Thessaloniki—AD vs. CN; and (**d**) Thessaloniki—FTD vs. CN. Solid lines denote the mean spectral feature trajectory for each condition, and shaded regions represent ±1 standard deviation across subjects for each epoch. The same number of epochs is displayed for all subjects within each comparison.

**Figure 5:**
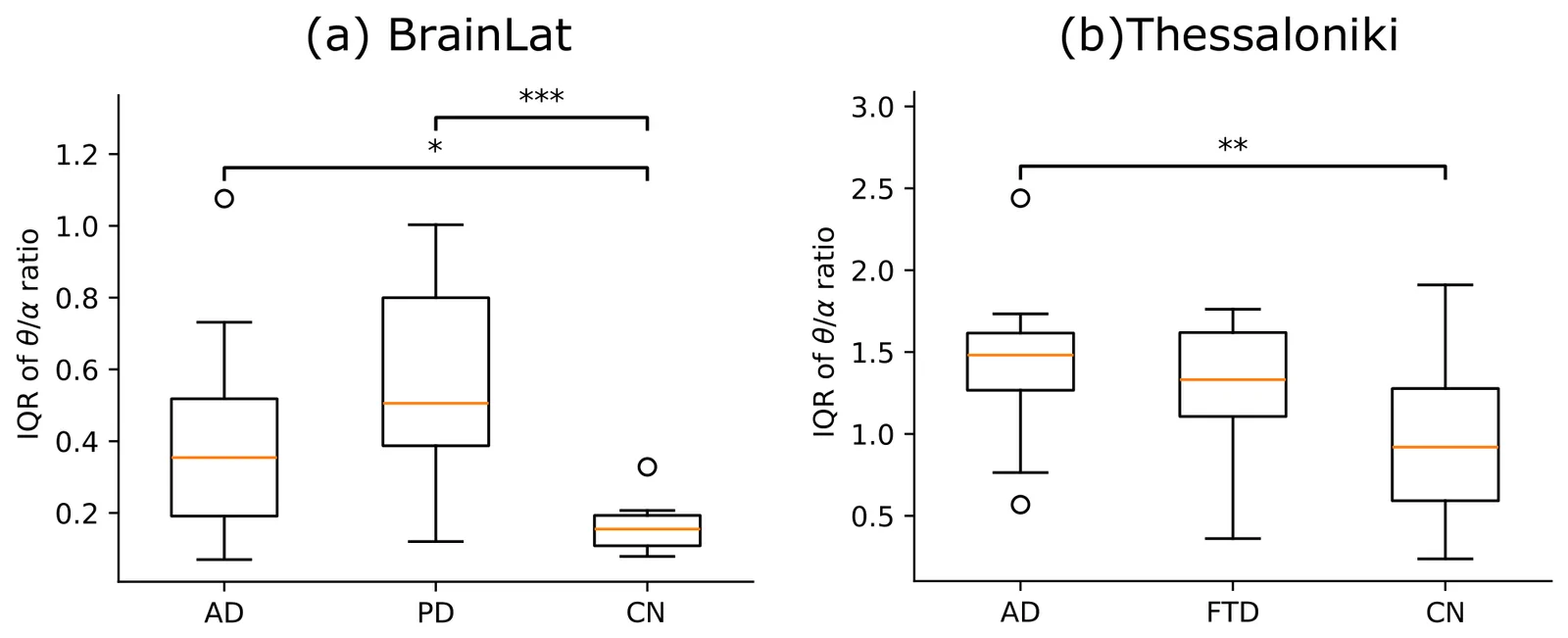
Temporal stability of the θ/α ratio. (**a**) BrainLat dataset and (**b**) Thessaloniki dataset. For each subject, temporal stability was quantified as the within-subject interquartile range (IQR) computed across consecutive epochs, with higher IQR values indicating lower temporal stability. Pairwise differences between each neurodegenerative condition and the corresponding CN group were assessed using the Mann–Whitney U test (* *p* < 0.05, ** *p* < 0.01, *** *p* < 0.001, Holm-adjusted).

**Figure 6:**
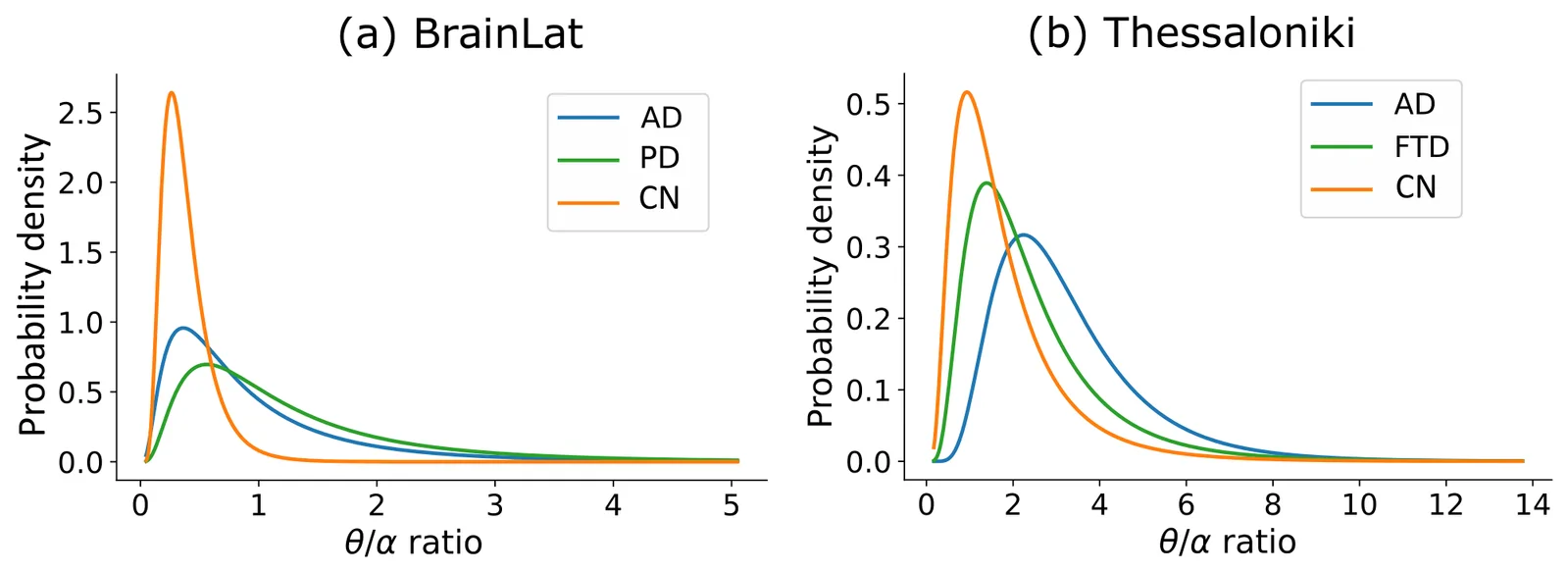
Lognormal models of the θ/α ratio across neurodegenerative conditions and normal aging for (**a**) the BrainLat dataset and (**b**) the Thessaloniki dataset. Curves represent maximum-likelihood lognormal fits to the complete positive empirical distribution of each group.

**Table 1:** Clinical and demographic characteristics of the datasets used for framework validation.

Dataset	Group	Age (Years)	Females (%)
BrainLat	CN (*n* = 12)	72.6±4.2	50
	AD (*n* = 19)	77.9±8.9	57.8
	PD (*n* = 22)	63.5±7.9	54.5
Thessaloniki	CN (*n* = 29)	67.9±5.3	62
	AD (*n* = 36)	66.4±7.8	66.6
	FTD (*n* = 23)	63.6±8	60.8

**Table 2:** Classification performance in two datasets using a Random Forest classifier under the LOSOCV strategy. Accuracy (Acc), sensitivity (Sen), specificity (Spe), precision (Prec), and F1-score (F1).

Dataset	Case	Acc (%)	Sen (%)	Spe (%)	Prec (%)	F1 (%)
BrainLat	AD vs. CN	67.7	73.6	58.3	73.6	73.6
	PD vs. CN	82.5	95.4	58.3	80.7	87.5
Thessaloniki	AD vs. CN	75.4	75	75.8	79.4	77.1
	FTD vs. CN	73.1	52.2	89.7	80	63.2

**Table 3:** Pairwise ANCOVA results testing the effects of Group and Age, and their interaction, on temporal stability of the θ/α ratio. * Holm-adjusted *p*-values for the pairwise Group effects within each dataset; *p*-values for Age and Group × Age are unadjusted.

Dataset	Comparison	Effect	*F*	*p*	$\eta_{p}^{2}$
BrainLat	AD vs. CN	Group	6.46	**<**0.05 *	0.20
		Age	0.00	0.94	<0.01
		Group × Age	0.18	0.67	<0.01
	PD vs. CN	Group	16.22	**<**0.001 *	0.34
		Age	8.29	**<** 0.01	0.21
		Group × Age	0.29	0.59	<0.01
Thessaloniki	AD vs. CN	Group	10.95	**<**0.01 *	0.24
		Age	0.19	0.66	0.01
		Group × Age	0.27	0.60	0.01
	FTD vs. CN	Group	0.54	0.47 *	0.02
		Age	0.56	0.46	0.02
		Group × Age	0.18	0.67	0.01

**Table 4:** Normalized log-likelihood ratio (*R*) and associated statistical significance (*p*) for model comparisons of the θ/α ratio across datasets and conditions.

Dataset	Group	$x_{\min}$	Lognormal vs. Power-Law R(p)	Lognormal vs. Exponential R(p)	Power-Law vs. Exponential R(p)
BrainLat	AD	0.99	5.01 ($5.42\times 10^{-7}$)	−5.78 ($7.56\times 10^{-9}$)	−5.77 ($7.73\times 10^{-9}$)
	PD	0.96	10.87 ($1.48\times 10^{-27}$)	2.76 ($5\times 10^{-3}$)	−17.61 ($1.95\times 10^{-69}$)
	CN	0.26	5.05 ($4.32\times 10^{-7}$)	1.15 (0.24)	−4.22 ($2.44\times 10^{-5}$)
Thessaloniki	AD	2.17	16.18 ($5.94\times 10^{-59}$)	5.25 ($1.44\times 10^{-7}$)	−24.26 ($4.41\times 10^{-130}$)
	FTD	1.91	10.61 ($2.54\times 10^{-26}$)	3.95 ($7.58\times 10^{-5}$)	−16.81 ($2.16\times 10^{-63}$)
	CN	1.61	10.92 ($8.51\times 10^{-28}$)	2.14 (0.03)	−15.12 ($1.14\times 10^{-51}$)

## Data Availability

The EEG datasets used in this study are publicly available at Synapse https://www.synapse.org/Synapse:syn51549340 (accessed on 23 September 2026) and OpenNeuro https://openneuro.org/datasets/ds004504 (accessed on 23 September 2026). The code and additional materials required to reproduce the analyses and results presented in this work are publicly available at Zenodo https://doi.org/10.5281/zenodo.22811463 (accessed on 23 September 2026) [38].

## Supplementary Materials

The following supporting information can be downloaded at https://www.mdpi.com/article/10.3390/diagnostics16193143/s1, Figure S1: Full SHAP beeswarm plots for the BrainLat dataset for both classification tasks: (a) Alzheimer’s disease (AD) vs. cognitively normal (CN), and (b) Parkinson’s disease (PD) vs. cognitively normal (CN); Figure S2: Full SHAP beeswarm plots for the Thessaloniki dataset for both classification tasks: (a) Alzheimer’s disease (AD) vs. cognitively normal (CN), and (b) frontotemporal dementia (FTD) vs. cognitively normal (CN); Figure S3: Inter-subject heterogeneity across consecutive epochs of the mean relative δ power for the AD vs. CN comparison in both datasets: (a) BrainLat and (b) Thessaloniki.; Figure S4: Histogram of the θ/α ratio across groups in the BrainLat dataset; Figure S5: Histogram of the θ/α ratio across groups in the Thessaloniki dataset; Table S1: Parameters of the lognormal models fitted to the θ/α ratio across groups in the BrainLat and Thessaloniki datasets.
