# Supplementary material for: An Interpretable Framework for Identifying EEG Spectral Biomarkers of Pathological Brain Aging
Source: Diagnostics (Basel). 2026 Sep 27;16(19):3143. doi: 10.3390/diagnostics16193143 (PMC13664103; doi:10.3390/diagnostics16193143)
Supplement: Supplementary file 1 [file diagnostics-16-03143-s001.zip › diagnostics-4515450-supplementary.pdf]

An Interpretable Framework for Identifying EEG Spectral Biomarkers of Pathological Brain Aging

Supplementary Material

A. SHAP-based analysis

Figures S1 and S2 present the SHAP beeswarm plots obtained for all classification tasks in the BrainLat and Thessaloniki datasets, respectively. These plots provide a comprehensive assessment of the full ranking of the 22 extracted spectral features and their contributions to the model predictions in terms of feature relevance and directionality in both datasets.

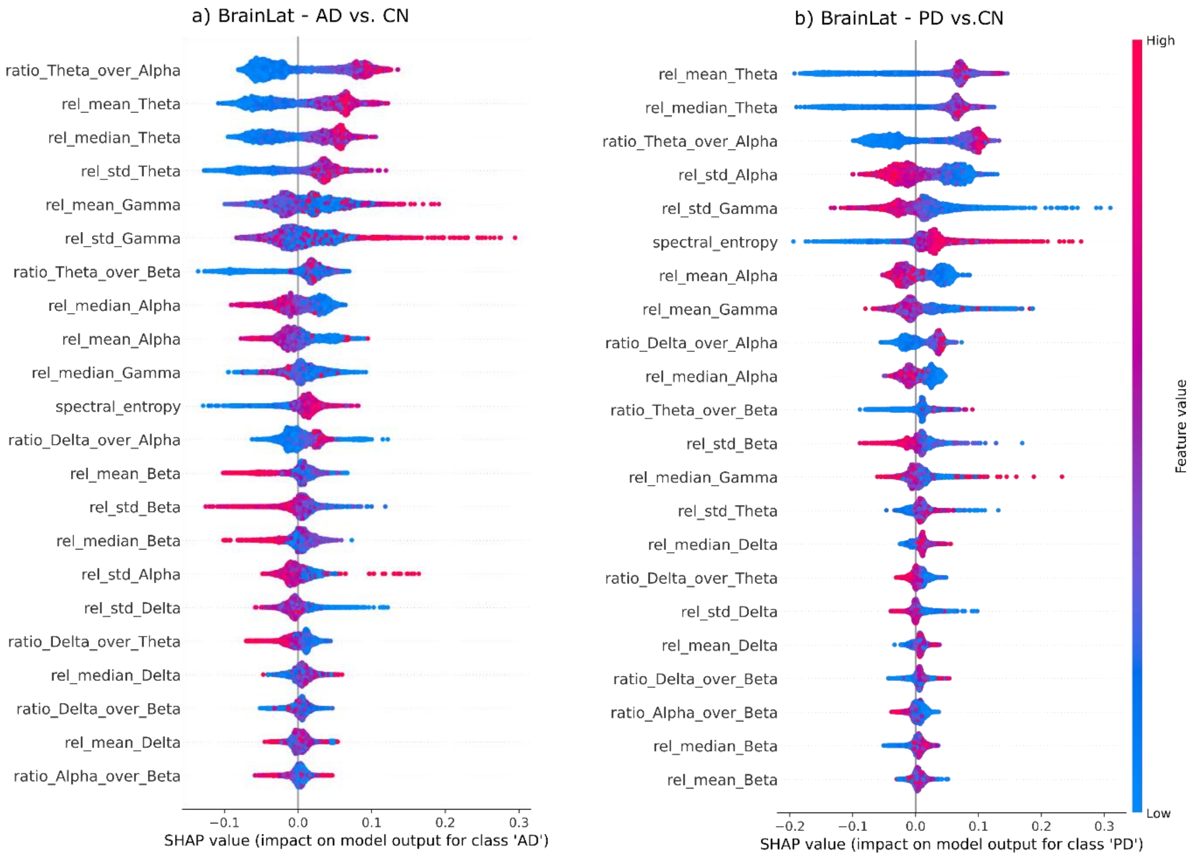

Figure S1. Full SHAP beeswarm plots for the BrainLat dataset for both classification tasks: (a) Alzheimer's disease (AD) vs. cognitively normal (CN), and (b) Parkinson's disease (PD) vs. cognitively normal (CN).

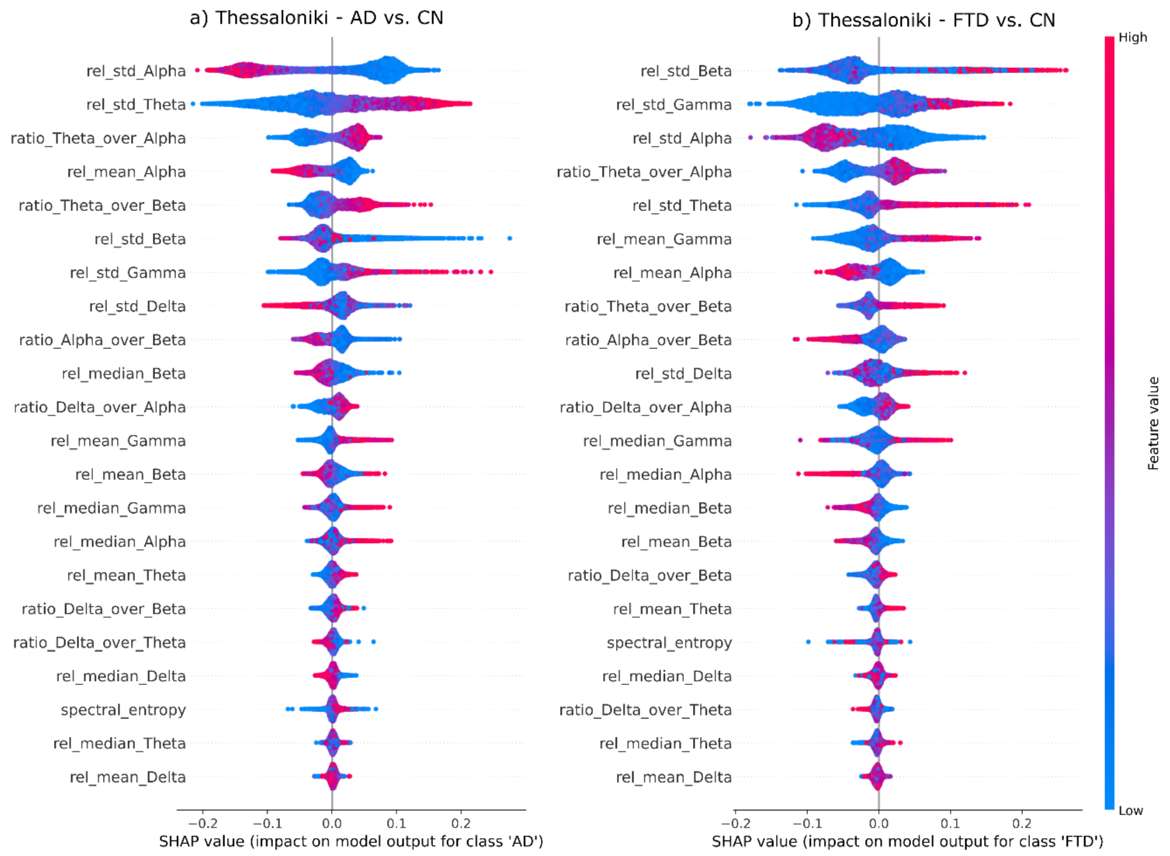

Figure S2. Full SHAP beeswarm plots for the Thessaloniki dataset for both classification tasks: (a) Alzheimer's disease (AD) vs. cognitively normal (CN), and (b) frontotemporal dementia (FTD) vs. cognitively normal (CN).

To further illustrate the relationship between feature relevance and inter-subject heterogeneity patterns, Figure S3 presents the inter-subject heterogeneity across consecutive epochs of the mean relative δ power, one of the least relevant features identified by the SHAP analysis in both datasets. Solid lines represent the group mean across epochs, and shaded regions denote ±1 standard deviation across subjects (inter-subject variability) at each epoch. In contrast to the θ/α ratio, which showed consistent relevance and marked differences in inter-subject heterogeneity between cognitively normal and neurodegenerative groups, the mean relative δ power exhibited largely overlapping profiles across conditions. This contrast indicates that the most relevant spectral features are not only discriminative for classification but also capture characteristic inter-subject heterogeneity associated with neurodegenerative conditions.

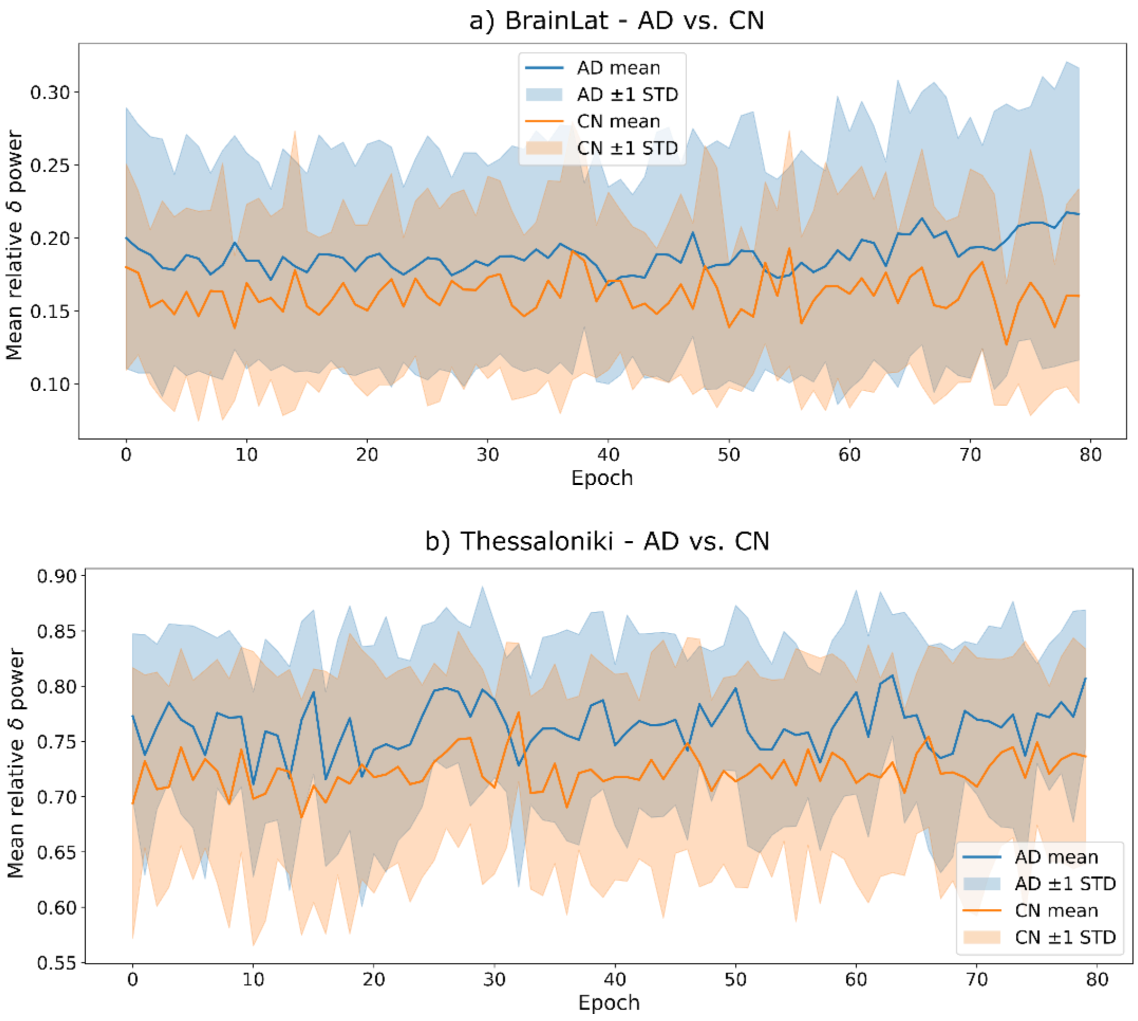

Figure S3. Inter-subject heterogeneity across consecutive epochs of the mean relative δ power for the AD vs. CN comparison in both datasets: (a) BrainLat and (b) Thessaloniki.

B. Modeling of spectral biomarkers

To characterize normative and pathological patterns in the key spectral feature identified by the SHAP analysis, probabilistic models were estimated for each diagnostic group. Figures S4 and S5 illustrate the empirical distributions of the θ/α ratio for the BrainLat and Thessaloniki datasets, respectively.

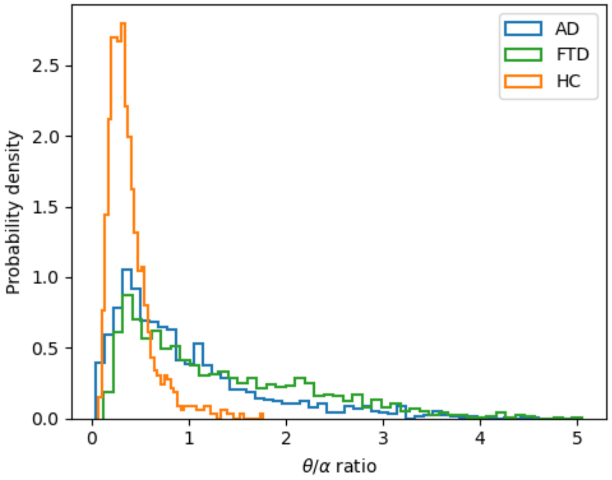

Figure S4. Histogram of the θ/α ratio across groups in the BrainLat dataset.

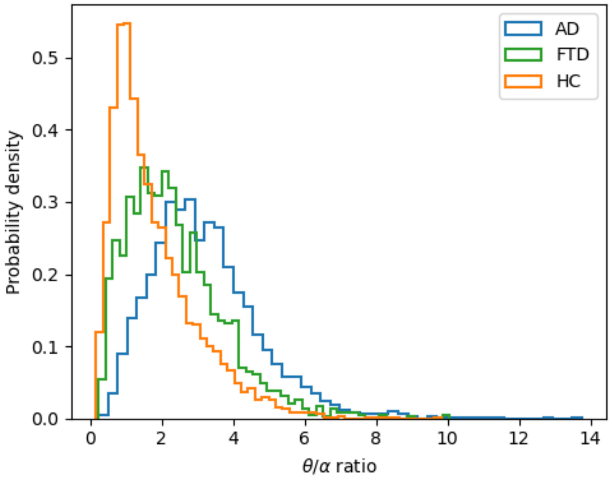

Figure S5. Histogram of the θ/α ratio across groups in the Thessaloniki dataset.

Although histogram visualizations provide an intuitive representation of feature distributions, they are sensitive to the choice of binning parameters.

B.1 Model comparison

To obtain a more robust characterization of the empirical distributions probabilistic models were considered. As an illustrative example, consider testing whether a power-law is a plausible hypothesis for the data. A random variable x is said to follow a power-law if it is drawn from a probability distribution:

$$p(x) \propto x^{-\alpha}$$

where α is the scaling parameter. In practice, power-law behavior typically holds only above some minimum value. For example, a continuous power-law distribution can be written as:

$$p(x) = Cx^{-\alpha}$$

where C is a normalization constant. Assuming $\alpha > 1$, the density diverges as $x \to 0$, so this expression cannot hold for all $x \geq 0$. Therefore, a lower bound x_{min} is introduced, such that the power-law behavior applies for $x \geq x_{min}$.

Before estimating the scaling parameter α, it is necessary to estimate x_{min} in order to discard all samples below this lower bound, leaving only those values for which the power-law model provides a valid approximation. Here, we estimated x_{min} by selecting the value that makes the empirical distribution of the data and the best-fitting power-law model as similar as possible for $x \geq x_{min}$.

To ensure a consistent basis for model comparison, the three candidate models (power-law, lognormal, and exponential) were fitted using the same estimated lower bound x_{min} (i.e., all fits were performed on data with $x \geq x_{min}$).

B.2 Biomarker distributions using a lognormal reference model

The lognormal distribution was used as a common reference model to characterize and compare biomarker distributions across groups. Its use as a reference model does not imply a statistically superior fit relative to the other candidate distributions. For each feature, the lognormal distribution was parameterized as $\ln(X) \sim \mathcal{N}(\mu, \sigma^2)$, where μ and σ denote the mean and standard deviation of $\ln(X)$, respectively. Table S1 reports the parameters of the lognormal models fitted for the θ/α ratio across all groups (the values are reported as μ and σ in log-space). Parameter estimation was performed using the full (non-truncated) data.

Table S1. Parameters of the lognormal models fitted to the θ/α ratio across groups in the BrainLat and Thessaloniki datasets.

Dataset	Group	μ	σ
BrainLat	AD	-0.35	0.79
	PD	-0.01	0.75
	CN	-1.07	0.48
Thessaloniki	AD	1.05	0.5
	FTD	0.7	0.31
	CN	0.37	0.66
